# Total DNA input is a crucial determinant of the sensitivity of plasma cell-free DNA EGFR mutation detection using droplet digital PCR

**DOI:** 10.18632/oncotarget.14390

**Published:** 2016-12-30

**Authors:** Yu Zhang, Yan Xu, Wei Zhong, Jing Zhao, Minjiang Chen, Li Zhang, Longyun Li, Mengzhao Wang

**Affiliations:** ^1^ Department of Respiratory Medicine, Peking Union Medical College Hospital, Peking Union Medical College, Chinese Academy of Medical Sciences, Beijing, China

**Keywords:** plasma cell-free DNA, droplet digital PCR, DNA concentration, epidermal growth factor receptor, advanced non-small cell lung cancer

## Abstract

We evaluated the use of droplet digital PCR (ddPCR) to detect plasma cell-free DNA (cfDNA) epidermal growth factor receptor (EGFR) mutations in advanced non-small cell lung cancer (NSCLC) patients. Compared with tumor-tissue-based detection, the sensitivity of ddPCR for detecting plasma cfDNA tyrosine kinase inhibitor (TKI)-sensitizing EGFR mutations was 61.3%, the specificity was 96.7%, and the consistency rate was 81.4% (?=0.605, 95% confidence interval: 0.501-0.706, p <0.0001). The sensitivity declined from 82.6% to 46.7% with decreasing cfDNA inputs (p=0.028). The plasma cfDNA concentration correlated with gender (males vs.females =11.69 ng/mL vs. 9.508 ng/mL; p=0.044), EGFR mutation status (tumor-tissue EGFR mutation-positive (EGFR M+) vs. EGFR mutation-negative (EGFR M-) = 9.61 ng/mL vs. 12.82 ng/mL; p =0.049) and specimen collection time (=2 years vs. >2 years=13.83 ng/mL vs. 6.575 ng/mL; p <0.001), and was greater in tumor-tissue EGFR M+ / plasma EGFR M+ patients than in tumor-tissue EGFR M+/plasma EGFR M- patients (11.61 vs. 7.73 ng/mL, respectively; p=0.003). Thus total cfDNA input crucially influences the sensitivity of plasma cfDNA EGFR mutation testing with ddPCR. Such analysis could be an effective supplemental test for advanced NSCLC patients.

## INTRODUCTION

Lung cancer has become the leading cause of cancer death [1]. It is well known that targeted therapy is an important treatment strategy for advanced non-small cell lung cancer (NSCLC) patients. Epidermal growth factor receptor (EGFR)-tyrosine kinase inhibitors (TKIs) are the most important targeted therapies for NSCLC, given their high clinical efficacy in NSCLC patients with TKI-sensitizing EGFR mutations [2–5]. However, tumor tissue samples are not always available for EGFR mutation detection in clinical practice. Rigorous clinical practice often requires the safe, effective, and dynamic real-time monitoring of the EGFR mutation status, and liquid biopsy may serve as a source of specimens [6, 7].

Plasma cell-free DNA (cfDNA) can be collected as a kind of liquid biopsy to reflect the tumor genotype to some extent [8, 9]. Plasma specimens can be obtained easily with minimal trauma, and can be monitored dynamically in real time [10, 11]. However, a strict requirement has been proposed for the detection of plasma cfDNA, due to the low cfDNA content and gene fragmentation in plasma. Many methods have been used to detect cfDNA EGFR mutations [11–16], and their sensitivities have ranged from 35.6% to 81.8% for EGFR mutation detection in cfDNA compared with tumor tissue.

Droplet digital PCR (ddPCR) is a digital PCR platform that allows the partitioning of input DNA into 20,000 droplets. This absolute quantification technique can be used to detect a mutant fraction as low as 0.001% [17, 18]. Zhu et al. [19] reported that the selective sensitivity of ddPCR was at least 0.04% in tests of EGFR mutation-positive cell DNA. To date, preliminary clinical studies have indicated that ddPCR is highly sensitive for detecting plasma cfDNA EGFR mutations [19–21].

We evaluated the feasibility of using ddPCR to detect EGFR mutations in plasma cfDNA from untreated advanced NSCLC patients in real-world clinical practice. We further analyzed the factors influencing the mutation analysis, and the correlation of EGFR mutation status with EGFR-TKI efficacy.

## RESULTS

### Clinical characteristics of patients

Table 1 lists the clinical characteristics of the 215 enrolled patients with NSCLC who were treatment-naïve at the time of plasma collection. The average age was 59.0 ± 11.5 years. Among these patients, 199 had adenocarcinoma, 2 had adenosquamous carcinoma, 4 had large-cell carcinoma, and 10 had NSCLC with an indefinite histological type. Subsequently, EGFR-TKIs were administered to 114 of these patients, 80 of whom were positive for EGFR TKI-sensitizing mutations (Exon 19 del and Exon 21 L858R) (EGFR M+) in tumor tissue, and 34 of whom were negative (EGFR M-). The EGFR-TKI treatments were as follows: gefitinib was administered in 94 cases, erlotinib in 18 cases, icotinib in 1 case, and afatinib in 1 case. An EGFR-TKI was used as the first-line treatment in 48 cases, second-line treatment in 49 cases, third-line treatment in 14 cases, and fourth-line treatment in 3 cases.

**Table 1:** Clinical Characteristics of 215 Patients with Non-squamous NSCLC

Item		Number of Patients	Percentage (%)
**Gender**	Male	127	59.1
	Female	88	40.9
**Age**	<60	108	50.2
	≥60	107	49.8
**Smoking history**	Yes	95	44.2
	No	120	55.8
**Disease staging**	Stage IIIB	36	16.7
	Stage IV	179	83.3
**Therapy received**	Chemotherapy	181	84.2
	EGFR-TKI treatment	114	53.0

### EGFR mutations in tumor tissues and plasma

Among the 215 tumor-tissue specimens, 102 EGFR gene mutations (46.5%) were found in 100 specimens, including 93 cases of Exon 19 del or Exon 21 L858R. We detected an Exon 19 del mutation in 44 patients (44.0%), Exon 21 L858R mutation in 47 patients (47.0%), Exon 18 G719X mutation in 2 patients (2.0%), Exon 20 S768I mutation in 2 patients (2.0%), Exon 20 insertion mutation in 2 patients (2.0%), Exon 21 L858R and Exon 20 T790M mutation in 1 patient (1.0%), and Exon 19 del and Exon 18 G719X mutation in 1 patient (1.0%).

Among the 215 plasma cfDNA samples, TKI-sensitizing Exon 19 del and Exon 21 L858R mutations were detected in 61 samples (28.4%), including 27 (12.6%) Exon 19 del mutations, 33 (15.3%) Exon 21 L858R mutations, and 1 (0.5%) Exon 19 del and Exon 21 L858R mutation. When the tumor-tissue-based EGFR gene results were regarded as the gold standard of diagnosis, the sensitivity of ddPCR in detecting plasma cfDNA EGFR Exon 19 del and Exon 21 L858R mutations was 61.3%, the specificity was 96.7%, and the consistency rate for both mutations was 81.4% (κ = 0.605, 95% confidence interval (CI): 0.501-0.706, P < 0.0001). Table 2 provides the detailed results.

**Table 2:** EGFR Exon 19 del and Exon 21 L858R Mutations Detected in Plasma cfDNA (ddPCR) Versus Paired Tumor Tissues (ARMS)

Plasma cfDNA	Tumor tissues		Total	Sensitivity	Specificity	Consistency rate	κ	P
	+	−						
**Exon19 del or** **Exon 21L858R**								
**+**	57	4	61	61.3%	96.7%	81.4%	0.605	<0.001
**−**	36	118	154					
**Total**	93	122	215					
**Exon 19del**								
**+**	26	2	28	57.8%	98.8%	90.2%	0.657	<0.001
**−**	19	168	187					
**Total**	45	170	215					
**Exon 21L858R**								
**+**	31	3	34	64.6%	98.2%	90.7%	0.701	<0.001
**−**	17	164	181					
**Total**	48	167	215					

### Univariate/Multivariate analysis of EGFR mutations in plasma and tissues

Univariate analysis was performed on the EGFR mutation status in plasma cfDNA samples and tumor tissues in accordance with gender, age, smoking history, pathological type, disease staging, and plasma cfDNA specimen collection time. Both the detection rate for plasma cfDNA EGFR mutations and the detection rate for tumor-tissue EGFR mutations correlated with gender and smoking history (Table 3). The plasma EGFR mutation detection rate was 23.8% among the 84 plasma specimens collected before May 31, 2012 (specimens collected > 2 years), and 31.3% among the 131 specimens collected after May 31, 2012 (specimens collected ≤ 2 years), indicating that there was no significant difference between these groups (P = 0.235).

**Table 3:** Univariate Analysis of EGFR Exon 19del and Exon 21 L858R Mutations and Clinical Characteristicsof 215 Patients with Non-squamous NSCLC

		Tumor tissue				Plasma cfDNA			
		EGFR Exon 19del and Exon 21 L858R Mutations		X^2^ Value	*P* Value	EGFR Exon 19del and Exon 21 L858R Mutations		X^2^ Value	*P* Value
		+	-			+	-		
**Gender**	Male	47	80	4.935	0.026	29	98	4.682	0.030
	Female	46	42			32	56		
**Age**	<60	51	57	1.391	0.238	33	75	0.509	0.476
	≥60	42	65			28	79		
**Smoking** **history**	Yes	29	66	11.237	<0.001	16	79	11.134	<0.001
	No	64	56			45	75		
**Pathological Type**	Adenocarcinoma	89	110	2.347	0.126	56	143	0.000	1.000
	Non-adenocarcinoma	4	12			5	11		
**Disease Staging**	Stage IIIB	13	23	0.899	0.343	11	25	0.101	0.750
	StageIV	80	99			50	129		
**Specimen Collection Time**	>2 years ago	33	51	0.885	0.347	20	64	1.412	0.235
	≤2 years ago	60	71			41	90		

Multivariate analysis of tumor-tissue mutations in the 215 cases revealed that only not smoking significantly influenced the tissue mutation test results (OR 2.487, 95% CI: 1.193-5.185, P = 0.015). Multivariate analysis of the six factors potentially influencing the plasma mutation analysis in the 215 cases indicated that both not smoking (OR 3.239, 95% CI: 1.408-7.450, P = 0.006) and having stage-IV NSCLC (OR 3.113, 95% CI: 1.109-8.735, P = 0.031) significantly influenced the mutation test results (Tables 4 and 5).

**Table 4:** Multivariate Analysis of Tissue EGFR Mutations and Clinical Characteristics

	OR	95% CI	Wald Value	*P* Value
**Age (≥60)**	0.859	0.486-1.520	0.271	0.603
**Gender (Female)**	0.993	0.478-2.061	0.000	0.984
**Pathological Type (Non- adenocarcinoma)**	0.492	0.148-1.634	1.343	0.247
**Smoking (No)**	2.487	1.193-5.185	5.907	0.015
**Disease Staging (Stage IV)**	1.371	0.633-2.973	0.640	0.424

**Table 5:** Multivariate Analysis of Plasma EGFR Mutations and Clinical Characteristics

	OR	95%CI	Wald Value	*P* Value
Age (≥60)	1.114	0.587-2.112	0.109	0.741
Gender (Female)	1.091	0.501-2.374	0.048	0.826
Pathological Type (Non- adenocarcinoma)	1.656	0.512-5.359	0.709	0.400
Smoking (No)	3.239	1.408-7.450	7.645	0.006
Disease Staging (Stage IV)	3.113	1.109-8.735	4.652	0.031
Specimen Collection Time (≤2 Years)	1.719	0.889-3.324	2.594	0.107

### Plasma cfDNA concentrations and cfDNA EGFR mutant alleles

ddPCR enables the absolute quantification of cfDNA templates. The total plasma cfDNA concentration (ng/mL plasma) and cfDNA template input per reaction (ng) were analyzed in our study. The median plasma cfDNA concentration of the 215 plasma samples was 11.09 ng/mL plasma (range, 1.01-2302.22), and the median amount of cfDNA per reaction was 3.24 ng (range, 0.29-672.24), for a median of 982 genome equivalents (GEs) per reaction (range 88-203,689).

**Figure 1:**
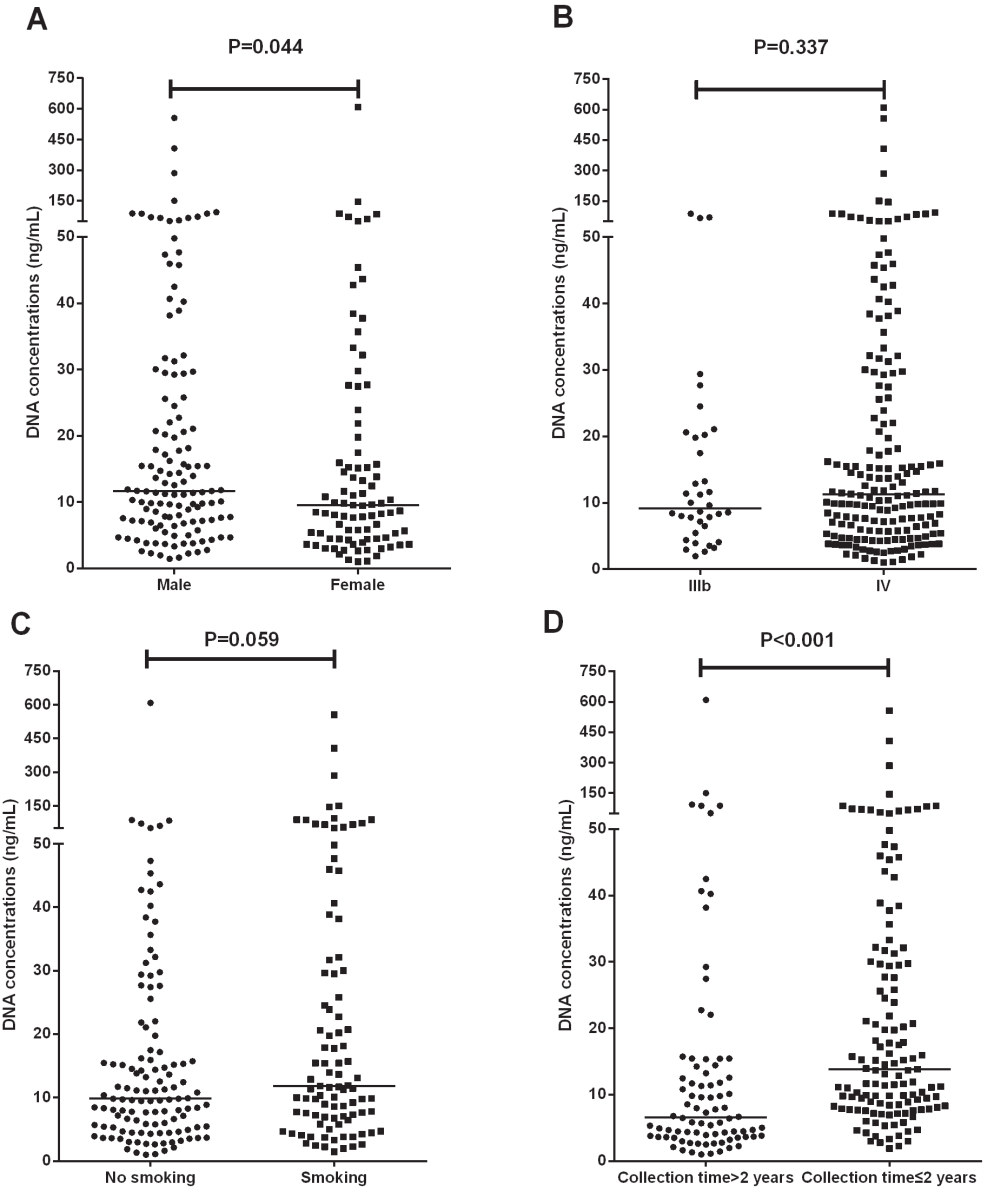
Analysis of the total cfDNA concentrations (ng/mL) of advanced non-squamous NSCLC plasma samples in accordance with gender, disease staging, smoking history, and plasma cfDNA specimen collection time

Univariate analysis of the cfDNA concentration (ng/mL) was performed in accordance with gender, age, smoking history, disease staging, and plasma cfDNA specimen collection time. The median plasma cfDNA concentration was higher for males than for females (11.69 ng/mL vs. 9.508 ng/mL, P = 0.044, Figure 1A). The plasma cfDNA concentration was slightly higher in smokers than in non-smokers, but the difference was not significant (median, 11.81 ng/mL vs. 9.829 ng/mL, P = 0.059, Figure 1C). The median plasma cfDNA concentration did not correlate with the disease stage (9.144 ng/mL for stage IIIB vs. 11.30 ng/mL for stage IV, P = 0.337, Figure 1B) or age (10.55 ng/mL for age < 60years vs. 11.12 ng/mL for ≥ 60years , P = 0.9188). The median plasma cfDNA concentration was clearly higher in specimens collected ≤ 2 years before the date of analysis than in those collected > 2 years earlier (13.83 ng/mL vs. 6.575 ng/mL, P < 0.001, Figure 1D).

**Figure 2:**
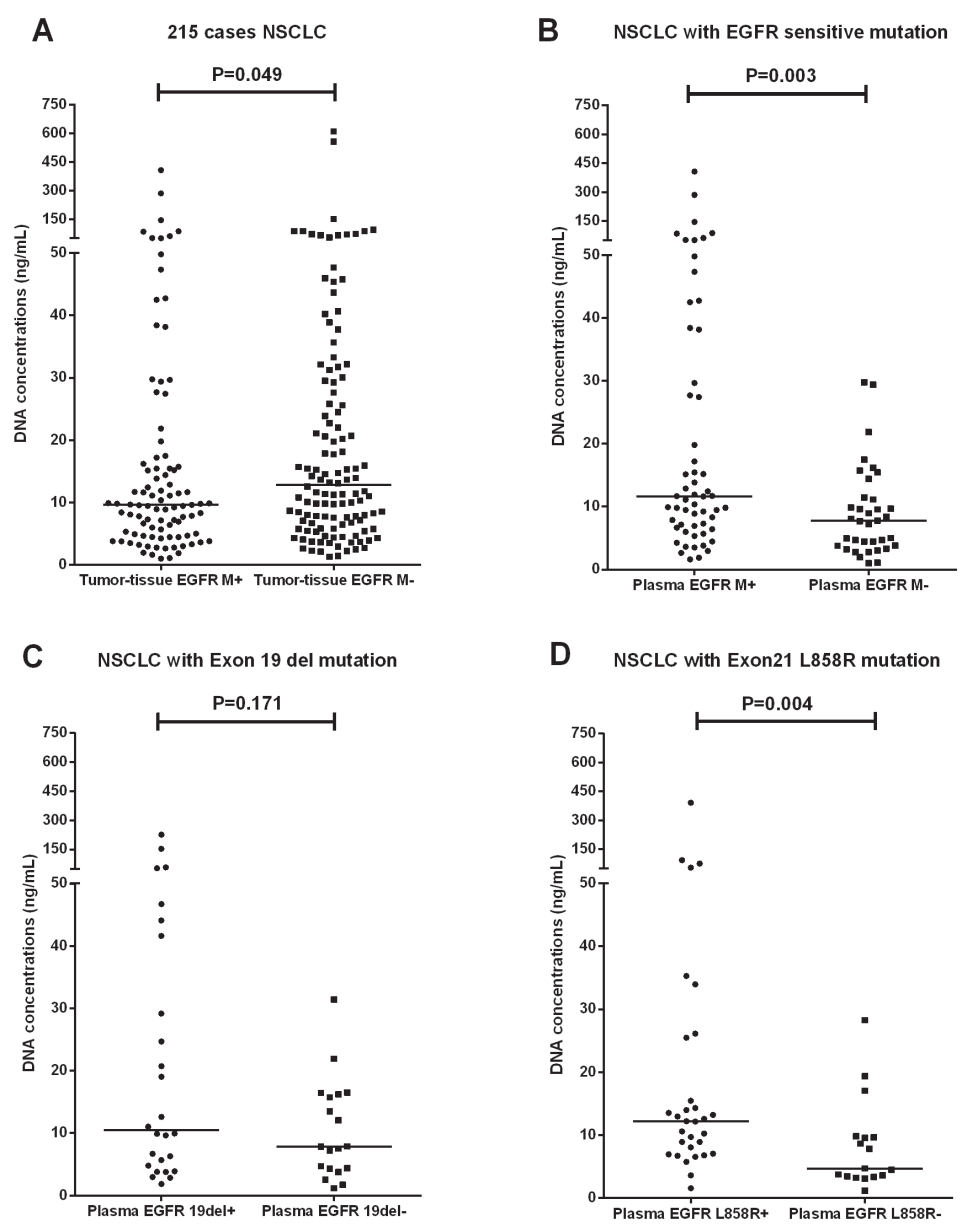
Total cfDNA concentrations (ng/mL) of advanced non-squamous NSCLC plasma samples **(A)** cfDNA concentrations of the 93 tumor-tissue EGFR M+ and 122 tumor-tissue EGFR M- patients (median, 9.61ng/mL vs. 12.82ng/mL, P=0.049); **(B)** cfDNA concentrations of the 57 tumor-tissue EGFR M+ / plasma EGFR M+ patients and 36 tumor-tissue EGFR M+ / plasma EGFR M- patients (median, 11.61ng/mL vs. 7.73 ng/mL, P=0.003); **(C)** cfDNA concentrations of the 26 tumor-tissue EGFR Exon 19 del M+ / plasma EGFR M+ patients and 19 tumor-tissue EGFR Exon 19 del M+ / plasma EGFR M- patients (median, 10.51 ng/mL vs.7.85 ng/mL, P=0.171); **(D)** cfDNA concentrations of the 31 tumor-tissue EGFR Exon 21 L858R M+ / plasma EGFR M+ patients and 17 tumor-tissue EGFR Exon 21 L858R M+ / plasma EGFR M- patients (median, 12.21 ng/mL vs.4.71ng/mL, P=0.004). EGFR M+: TKI-sensitizing epidermal growth factor receptor mutation (Exon 19del and Exon 21L858R)-positive; EGFR M-: TKI-sensitizing epidermal growth factor receptor mutation (Exon 19del and Exon 21L858R)-negative.

The median plasma cfDNA concentrations of the 93 tumor-tissue EGFR M+ and 122 tumor-tissue EGFR M- patients were 9.61 ng/mL (range 1.01-406.91) and 12.82 ng/mL (range 1.32-2302.22), respectively; this difference was statistically significant (P = 0.049) (Figure 2A). Among the 93 tumor-tissue EGFR M+ patients, the median cfDNA concentration of the 57 plasma EGFR M+ patients was 11.61 ng/mL (range 1.63-406.91), while that of the 36 plasma EGFR M- patients was 7.73 ng/mL (range 1.01-29.76); this difference was statistically significant (P = 0.003) (Figure 2B). The same results were found when Exon 21 L858R M+/M- plasma samples were compared (median, 12.21 ng/mL vs. 4.71 ng/mL, P = 0.004, Figure 2D), but not when Exon 19 del M+/M- plasma samples were compared (median, 10.51 ng/mL vs. 7.85 ng/mL, P = 0.171, Figure 2C)

Quantitation of cfDNA EGFR mutant alleles was also performed for the 57 plasma EGFR M+ patients. The median abundance of EGFR mutations was 4.15% (range 0.05%-74.5%). The median DNA concentration did not differ significantly between the high-abundance ( > 4.15%) group and the low abundance ( ≤ 4.15%) group (14.49 ng/mL vs. 10.37 ng/mL, P = 0.343).

### Relationship between plasma cfDNA input and plasma cfDNA EGFR mutation detectability

**Figure 3:**
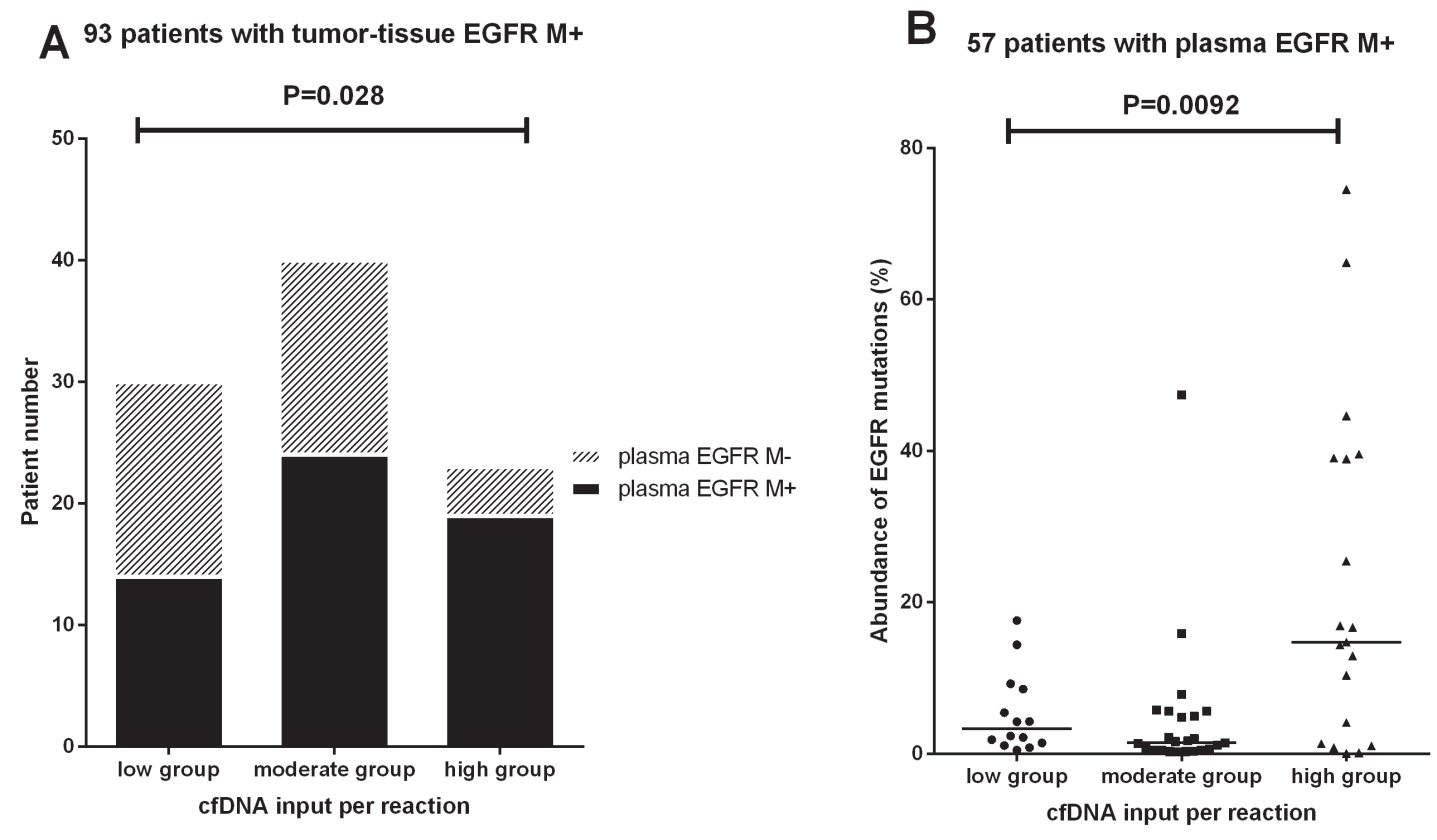
Relationship between plasma cfDNA input and plasma cfDNA EGFR mutation detectability **(A)**Plasma EGFR mutation results of 93 tumor-tissue EGFR M+ patients in different cfDNA input groups.**(B)** Abundance of EGFR mutations in 57 plasma EGFR M+ patients in different cfDNA input groups. High group:≥5ng cfDNA input per reaction; Moderate group: 2-5ng cfDNA per reaction; Low group: <2ng cfDNA input per reaction.

To better understand the plasma false-negative results in tumor-tissue EGFR M+ patients, we assessed the quantity and quality of cfDNA from the 93 tumor-tissue EGFR M+ patients (true positives) according to the cfDNA input. The sensitivity was 82.6% for the 23 cases with cfDNA inputs of ≥ 5 ng per reaction, 60% for the 40 cases with 2-5 ng cfDNA per reaction, and 46.7% for the 30 cases with < 2ng cfDNA per reaction. The difference among the three groups was statistically significant (P = 0.028, Figure 3A).

The median abundance of EGFR mutations in the high cfDNA input group ( ≥ 5 ng/reaction) was 14.5% (range 0.05%-74.5%), which was higher than those in the moderate group (1.5% for 2-5ng cfDNA per reaction) and the low group (3.3% for < 2 ng cfDNA per reaction). The difference among the three groups was statistically significant (P = 0.0092, Figure 3B).

### Correlation of plasma and tumor-tissue EGFR gene mutations with targeted drug efficacy

**Figure 4:**
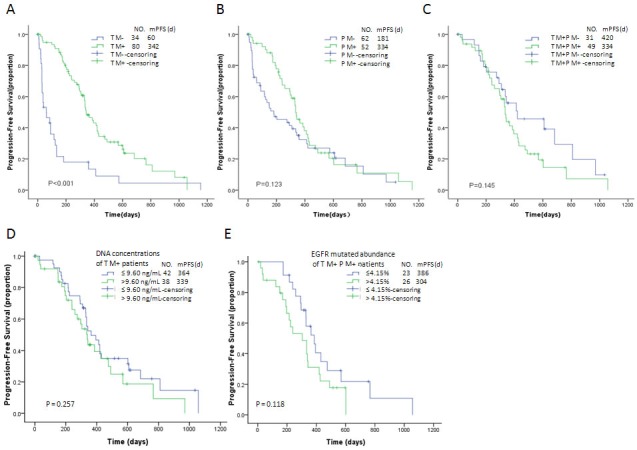
Kaplan-Meier curves of progression-free survival for tumor-tissue EGFR M+/M- patients (by ARMS) treated with EGFR-TKIs (**A**), for plasma EGFR M+/M- patients (by ddPCR) treated with EGFR-TKIs (**B**), for tumor-tissue EGFR M+ patients (by ARMS) who were either plasma EGFR M+ or EGFR M- (by ddPCR) and treated with EGFR-TKIs (**C**), for tumor-tissue EGFR M+ patients with high cfDNA concentrations (>9.6ng/mL) or low cfDNA concentrations (≤9.6ng/mL) treated with EGFR-TKIs (**D**), and for tumor-tissue EGFR M+ / plasma EGFR M+ patients with high EGFR mutation abundance(>4.5%) or low EGFR mutation abundance (≤4.5%)and treated with EGFR-TKIs **(E).** * EGFR M+: TKI-sensitizing epidermal growth factor receptor mutation (Exon 19del and Exon 21L858R)-positive. EGFR M-: TKI-sensitizing epidermal growth factor receptor mutation (Exon 19del and Exon 21L858R)-negative TM +: tumor tissue EGFR M+ TM -: tumor tissue EGFR M- TM+ censoring: tumor tissue EGFR M+ censoring TM- censoring: tumor tissue EGFR M- censoring PM +: plasma EGFR M+ PM -: plasma EGFR M- PM+ censoring: plasma EGFR M+ censoring PM- censoring: plasma EGFR M- censoring TM+PM-: tumor tissue EGFR M+ and plasma EGFR M- TM+PM+: tumor tissue EGFR M+ and plasma EGFR M+ TM+PM-censoring: Censoring data from the tumor-tissue EGFR M+ / plasma EGFR M-group TM+PM+censoring: Censoring data from the tumor-tissue EGFR M+ / plasma EGFR M+ group

Among the 114 patients treated with EGFR-TKIs, 80 (70.1%) were positive for tumor-tissue TKI-sensitizing EGFR mutations, including 45 Exon 21 L858R mutations, 33 Exon 19 del mutations, 1 Exon 19 del and Exon 18 G719X mutation, and 1 Exon 18 G719X mutation, whereas 32 (28.1%) were negative for tumor-tissue EGFR mutations and 2 (1.8%) were TKI-resistanting EGFR mutations(1 Exon 21 L858R/Exon 20 T790M mutation and 1 Exon 20 insertion mutation). In total, 49 patients (43.0%) were both tumor-tissue and plasma cfDNA EGFR M+; 31 patients (27.2%) were tumor-tissue EGFR M+ but plasma cfDNA EGFR M-; 3 patients (2.6%) were tumor-tissue EGFR M- but plasma cfDNA EGFR M+; and 31 patients (27.2%) were both tumor-tissue and plasma cfDNA EGFR M-. After EGFR-TKI treatment, the patients who were TKI-sensitizing EGFR mutations had a median progression-free survival (PFS) of 342 days (95% CI: 290.6-393.4), and those who were tumor-tissue EGFR M- had a median PFS of 60 days (95% CI: 0.0-123.8); this difference was statistically significant (P < 0.001, Figure 4A). After EGFR-TKI treatment, the median PFS of the 52 plasma cfDNA EGFR M+ patients was 334 days (95% CI: 297.5-370.5), whereas that of the 62 cfDNA EGFR M- patients was 181 days (95% CI: 37.6-324.4); however, this difference was not statistically significant (P = 0.123, Figure 4B).

Among the 80 tumor-tissue TKI-sensitizing EGFR M+ patients, the median PFS of the 49 plasma EGFR M+ patients was 334 days (95% CI: 317.0-351.0), whereas that of the 31 cfDNA EGFR M- patients was 420 days (95% CI: 100.1-739.9), indicating no significant difference between the groups (P = 0.145, Figure 4C). When these 80 patients were subdivided into two groups based on the median plasma cfDNA concentrations (low group: ≤ 9.60 ng/mL, high group: > 9.60ng/mL), the median PFS did not differ significantly between the two groups (364 days vs. 339 days, P = 0.257, Figure 4D).

Moreover, the 49 patients who were both tumor-tissue and plasma EGFR M+ were subdivided into two groups based on the median abundance of EGFR mutations (low group: ≤ 4.15%, high group: > 4.15%). The median PFS did not differ between these two groups, either (386 days vs. 304 days, P = 0.118, Figure 4E).

The three patients who were tumor-tissue EGFR M- but plasma cfDNA EGFR M+ had PFS times of 133, 410, and 1,153 days, respectively, after EGFR-TKI treatment.

## DISCUSSION

Plasma cfDNA could be an effective source of specimens for EGFR detection in NSCLC patients, because it is easily accessible and can be evaluated repeatedly at different times. However, there is no consensus as to which method is the best for detecting plasma cfDNA EGFR mutations. The ddPCR assay is reported to be highly sensitive and specific in detecting plasma cfDNA EGFR mutations. Our study revealed that the sensitivity of ddPCR was 61.3% when tumor-tissue EGFR mutation was used as the gold standard. While ddPCR can accurately determine the quantity and quality of cfDNA, this study revealed for the first time that the cfDNA concentration/input is a crucial factor affecting the sensitivity of cfDNA EGFR mutation tests.

The detection rate of EGFR mutations in the tumor tissues in this study was 46.5%. Univariate analysis demonstrated that the mutation incidence was high in female and non-smoking patients, and multivariate analysis also indicated that not smoking correlated with tumor-tissue EGFR mutation status (OR 2.487, 95% CI: 1.193-5.185, P = 0.015). All these results are consistent with previous findings [4, 22, 23]. Zhu et al. [19] found that the plasma EGFR mutation testing sensitivity of ddPCR was greater than 80%, and the specificity was approximately 95.8-98.4%, compared with ARMS testing of matched tumor tissues. In our study, the specificity for plasma EGFR testing by ddPCR was 96.7-98.8%, similar to the results reported by Zhu et al. However, the sensitivity in our study was lower than those previously reported for the ddPCR method [19, 20, 24] and the Cobas 4800 blood test [25], but higher than those reported for ARMS [12, 26] or other tests [27]. More than 200 NSCLC patients were enrolled in our study, and the blind method was adopted, so our study was closer to a clinical practice situation. In a large, multinational, non-interventional, real-world EGFR mutation analysis study (IGNITE [NCT01788163]), the sensitivity for plasma EGFR testing was found to be 46.9%, demonstrating that the sensitivity of plasma EGFR testing in the real world may be lower than it is in laboratory settings.

In order to understand the possible reasons for the relatively low sensitivity in our study, we analyzed the plasma cfDNA concentration and the cfDNA template input per reaction. The median plasma cfDNA concentration of tumor-tissue EGFR M+ patients was lower than that of EGFR M- patients (9.61 ng/mL vs. 12.82 ng/mL, P = 0.049), possibly indicating that less cfDNA is released from the tumors of the EGFR M+ patients than from those of the EGFR M- patients. Interestingly, when the plasma specimens of the 93 tumor-tissue EGFR M+ patients were divided into two groups according to the results of the cfDNA test, the median cfDNA concentration of the cfDNA EGFR M+ group was much higher than that of the cfDNA EGFR M- group (11.61 ng/mL vs. 7.73 ng/mL, P = 0.003). Thus, the cfDNA concentration correlated with the sensitivity of the EGFR mutation test in plasma.

Other studies have analyzed the effect of the cfDNA concentration on the EGFR mutation detection rate. Oxnard et al. [28] adopted a second-generation sequencing method to analyze the genotypes of NSCLC patients, and assessed the cfDNA quality and quantity by evaluating the human long interspersed element 1 (LINE-1) concentration. The authors obtained a cfDNA EGFR detection sensitivity of 50% when the LINE-1 concentration was lower than the median value, but 81% when the concentration was higher than the median value.

In the interpretation of cfDNA mutation results, the cfDNA concentration must be considered, just as tumor tissue must first be evaluated by a pathologist before molecular testing. In our study, the sensitivity of the cfDNA test was 82.6% in patients with cfDNA inputs of ≥ 5 ng per reaction. However, only 23 of 93 patients (24.7%) had cfDNA inputs of ≥ 5 ng per reaction, while 30 of 93 patients (32.3%) had cfDNA inputs of < 2ng per reaction. We used 2 mL of plasma from each patient for our analyses. In real-world settings, 4 mL of plasma might be better for cfDNA testing, because doubling the concentration of cfDNA extracted should double the cfDNA input and thus increase the sensitivity. Moreover, the median plasma cfDNA concentration for specimens collected ≤ 2 years before the analysis was obviously higher than that of specimens collected > 2 years before the analysis (13.83 ng/mL vs. 6.575 ng/mL, P < 0.001). Thus, the plasma cfDNA degraded gradually, despite being stored at −80°C - an inevitable defect of a retrospective study. Prospective studies or retrospective studies of plasma specimens collected ≤ 2 years before analysis will be better for further research.

This study further investigated the relation between the efficacy of EGFR-TKIs and mutation status. The median PFS of tumor-tissue EGFR M+ patients was significantly higher than that of EGFR M- patients, consistent with the IPASS results reported by Mok et al. [29]. The median PFS of plasma EGFR M+ patients (334 days) was longer than that of plasma EGFR M- patients (181 days), but the difference was not statistically significant (P = 0.123, Figure 2B). The reason for this was clearly that some tumor-tissue EGFR M+ patients were detected as false negatives in cfDNA tests. This is the shortcoming of cfDNA testing, even when ddPCR is applied [11].

Among 80 tumor-tissue EGFR M+ patients treated with EGFR-TKIs, the median PFS of plasma EGFR M+ patients was shorter than that of plasma EGFR M- patients, but the difference was not significant (334 days vs. 420 days, P = 0.145). Li et al. [30] found that EGFR-TKI treatment of tumor-tissue EGFR M+ patients prolonged the PFS of plasma EGFR M- patients compared with plasma EGFR M+ patients, though the result was not statistically significant (19.7 months vs. 11.0 months, P = 0.102). Furthermore, Lam et al. [31] found that both the PFS and the OS of plasma EGFR M+ patients were shorter than those of plasma EGFR M- patients. In our study, the cfDNA concentration of plasma EGFR M+ patients was significantly higher than that of EGFR M- patients. A high cfDNA concentration may indicate a large tumor burden [32] and poor prognosis. This result warrants validation in further studies.

Four patients (4/215, 1.9%) who were tumor-tissue EGFR M- but plasma EGFR M+ were enrolled in our study, and three of them benefited from EGFR-TKI treatment. This phenomenon may be attributed to tumor heterogeneity or false negative results from tumor-tissue detection methods. Follow-up data from the three patients who benefited from EGFR-TKI treatment demonstrated that either cfDNA or tumor-tissue EGFR mutation positivity can be treated as a true positive and can predict the efficacy of EGFR-TKI treatment. Patients who are tumor-tissue EGFR M- but cfDNA EGFR M+ have been described in many studies [26, 27, 33, 34]. In these studies (including our study), 155 patients were detected as cfDNA EGFR M+ and tumor-tissue EGFR M-, among a total of 3834 patients. Thus, the frequency of tumor-tissue EGFR M- and plasma cfDNA EGFR M+ patients is 4.0% (155/3834), which is higher than the frequencies of tumor tissue rare mutations such as ROS1, RET, BRAF, etc [35, 36]. Thus, while plasma is an inadequate substitute for tumor tissue in EGFR mutation tests, plasma testing could be used for tumor-tissue negative patients as an effective supplemental test.

This study has a few limitations. First, this was a retrospective study, so prospective studies are needed in future, especially for the evaluation of EGFR-TKIs. Second, 2 mL of plasma was collected from all patients for analysis, but 4 mL might be a better volume for the purpose of achieving a high total cfDNA input. Third, the use of fresh plasma samples or plasma specimens collected ≤ 2 years before analysis might increase the cfDNA concentration. Finally, the cfDNA concentration should be analyzed first, and additional plasma should be used for the detection of EGFR mutations in patients with low cfDNA concentrations.

In summary, our study indicated that ddPCR can be used for plasma cfDNA EGFR mutation detection, and plasma cfDNA EGFR mutation analysis could be an effective supplemental test for tumor-tissue EGFR M- patients. Moreover, we found that the cfDNA concentration and cfDNA input are crucial factors influencing the sensitivity of cfDNA EGFR mutation testing by ddPCR, and that less cfDNA is released from the tumors of EGFR M+ patients than from those of EGFR M- patients. Consequently, a larger volume of fresh plasma should be used for cfDNA detection in real-world practice. The applicability of ddPCR for cfDNA mutation testing in clinical practice requires further evaluation.

## MATERIALS AND METHODS

### Study subjects

For this study, we retrospectively enrolled 215 patients who were treated at Peking Union Medical College Hospital (PUMCH) from July 9, 2009 to May 31, 2014. All newly diagnosed NSCLC patients and corresponding treatment-naïve plasma specimens were numbered in the PUMCH lung cancer clinical database and tumor/plasma specimen pool, respectively. The eventual treatment and follow-up data were subsequently added to the clinical database. The NSCLC patients in our clinical database who met the inclusion criteria were enrolled in this study retrospectively. The following inclusion criteria were considered: age ≥ 18 years; pathological diagnosis of non-squamous NSCLC; disease stage IIIB or IV; measurable lesions in radiographic findings; EGFR mutation status of tumor tissue which had been tested by ADx-ARMS in clinical practice; complete clinical data; good follow-up; and treatment naïveté at the time of plasma collection (after which the patients could receive anti-cancer treatment). This study was approved by the Ethics Committee of PUMCH, and the project approval number was S-675. All patients signed the informed consent form for specimen collection, clinical information collection, and biomarker analysis.

### Study design

This study was designed as a single-blind test. EGFR mutations in plasma cfDNA were detected by the ddPCR method. Clinical data that were collected included gender, age, smoking history, tumor pathological type, disease staging according to 7th version released by the Union for International Cancer Control and International Association for the Study of Lung Cancer in 2009, EGFR mutation status of tumor tissues, treatment, and survival. The efficacy of EGFR-TKI treatment was evaluated in accordance with RECIST1.1. The last follow-up visit was on Dec 31, 2015. The median follow-up time was 22.5 months.

### Plasma cfDNA EGFR mutation (Exon 19 del and Exon 21 L858R) detection by ddPCR

Peripheral venous blood samples (10 mL) from untreated NSCLC patients were collected into EDTA anticoagulant tubes after patients signed informed consent forms. The tubes were centrifuged at 3000 rpm for 10 min at 4°C for plasma isolation. Subsequently, the plasma specimens were transferred to Eppendorf tubes and stored at −80°C until cfDNA extraction. Plasma cfDNA was extracted from 2 mL of plasma from each patient with a QIAamp Circulating Nucleic Acid Kit (Qiagen, Hilden, Germany), in accordance with the manufacturer's instructions. Extracted cfDNA from each plasma sample was eluted in 50 μL of Tris-EDTA buffer and stored at −20°C until further analysis.

For PCR amplification, 7.3 μL of plasma cfDNA obtained in the above steps was loaded into the reaction mix. The final 20 μL of the TaqMan PCR reaction mixture was prepared as described previously [19]. Each assembled ddPCR reaction mixture and 70 μL of droplet generation oil were loaded into a droplet generator (Bio-Rad Laboratories, Hercules, CA, USA) to generate an emulsion of ~20,000 droplets. The PCR thermal profile was the same as described previously [19]. Four wells of negative controls with human reference genomic DNA, two wells of positive controls with 1:2500 ratios of mutant alleles to wildtype alleles, and two wells of non-template controls were included in every run. After PCR amplification, the results were read with a QX100 Droplet Reader (Bio-Rad Laboratories) as instructed. Analysis of the ddPCR data for allele calling was performed with QuantaSoft version 1.3.2.0 (Bio-Rad Laboratories).

As described in a previous paper [19], the plasma cfDNA input (I, in the unit of GEs/reaction) was calculated with the following equation:

I (GEs/reaction) = -ln(1- p)/V ×1000 × 20

(p = positive droplets, V = 0.91 nL)

The plasma cfDNA input (W, in the unit of ng/reaction) was calculated as follows:

W (ng/reaction) = I(GEs/reaction)/303(GEs/ng)

The plasma cfDNA concentration (C, in the unit of ng/mL plasma) was calculated as follows:

C (ng/mL plasma) = W(ng/reaction)/V1×V2/V3

(V1 = 7.3 µL cfDNA/reaction; V2 = 50 µL extracted cfDNA; V3 = 2 mL plasma)

As described in a previous paper [19], the fractions of EGFR Exon 21 L858R mutants (F1) and EGFR Exon 19 del mutants (F2) were calculated as follows:

F1 = I (FAM)/( I (FAM)+I (VIC) )

F2 = I (FAM)/I (VIC)

(I (FAM) = mutant DNA templates; I (VIC) = wild-type DNA templates)

### Statistical analysis

The tumor-tissue EGFR mutation status was considered as the gold standard for assessing the sensitivity and specificity of plasma cfDNA results. The consistency between plasma cfDNA and tumor-tissue results was assessed by Cohen's κ test. The difference between groups was tested with Pearson's chi-square method. The DNA concentrations of two independent specimens of plasma sample DNA were tested with the rank sum test. Logistic regressions were adopted for multivariate analysis of EGFR mutations in plasma and tumor tissues. The period from the first day of EGFR-TKI application to the day of disease progression was deemed as the PFS time. PFS curves were estimated by the Kaplan-Meier method with a log-rank test. Statistical analysis was performed with SPSS for Windows (SPSS version 17.0, SPSS Inc., Chicago, IL, USA) and GraphPad Prism (GraphPad Software, San Diego, CA, USA). A two-tailed P < 0.05 was considered significant.
